# Noncoder: a web interface for exon array-based detection of long non-coding RNAs

**DOI:** 10.1093/nar/gks877

**Published:** 2012-09-24

**Authors:** Pascal Gellert, Yuliya Ponomareva, Thomas Braun, Shizuka Uchida

**Affiliations:** ^1^Max Planck Institute for Heart and Lung Research, Ludwigstr. 43, 61231 Bad Nauheim, Germany and ^2^Institute for Cardiovascular Regeneration, Centre for Molecular Medicine, Goethe University, Theodor Stern-Kai 7, 60590 Frankfurt, Germany

## Abstract

Due to recent technical developments, a high number of long non-coding RNAs (lncRNAs) have been discovered in mammals. Although it has been shown that lncRNAs are regulated differently among tissues and disease statuses, functions of these transcripts are still unknown in most cases. GeneChip Exon 1.0 ST Arrays (exon arrays) from Affymetrix, Inc. have been used widely to profile genome-wide expression changes and alternative splicing of protein-coding genes. Here, we demonstrate that re-annotation of exon array probes can be used to profile expressions of tens of thousands of lncRNAs. With this annotation, a detailed inspection of lncRNAs and their isoforms is possible. To allow for a general usage to the research community, we developed a user-friendly web interface called ‘noncoder’. By uploading CEL files from exon arrays and with a few mouse clicks and parameter settings, exon array data will be normalized and analysed to identify differentially expressed lncRNAs. Noncoder provides the detailed annotation information of lncRNAs and is equipped with unique features to allow for an efficient search for interesting lncRNAs to be studied further. The web interface is available at http://noncoder.mpi-bn.mpg.de.

## INTRODUCTION

Originally, transcripts were considered mostly as templates for proteins. Therefore, it came as a surprise that a low number of protein-coding genes were identified when the first human draft genome was published. Over a decade of studies (mostly owing to the technical advances in high-throughput technologies, e.g. tiling microarrays and RNA-Seq) revealed that the transcriptome of eukaryotes is much more complex than originally hypothesized. Nowadays, it is generally accepted that a high number of non-coding RNAs are present in a cell, which exceeds the number of protein-coding genes (1). One of the classes of non-coding RNAs is called ‘long non-coding RNAs (lncRNAs)’, which have been defined solely by their size of >200 nucleotides (2). To further specify the members of this group, they are often classified into several sub-categories by their genomic proximity to protein-coding genes: sense (overlapping on the same strand), antisense (overlapping on the opposite strand), bidirectional (encoded on the opposite strand of a protein-coding gene in a head-to-head orientation), intronic (entirely within an intron) and intergenic (in far distance to protein-coding genes, often called long intergenic non-coding RNAs or lincRNAs) (2). LncRNAs share some similarity with protein-coding genes as they can be spliced into different isoforms (3,4), and are often 5′-capped and/or polyadenylated (5–7). In contrast, their expression levels are generally low (1), and they are not well conserved among species (8). These findings have led to an ongoing debate about their functionalities (9–11). One evidence for the function of lncRNAs is the regulation of their expressions by transcription factors (12,13). It could be shown that lncRNAs are differentially expressed during the development of an organism (12) and in a tissues-specific manner, even to a more extreme than protein-coding genes (4,14). LncRNAs are also suspected to play an important role in diseases as their expression changes between disease and non-disease states (2,13).

The Affymetrix GeneChip Exon 1.0 ST arrays (exon arrays) contain ∼5.4 and ∼4.7 million probes on the human and mouse versions, respectively. They are designed to measure the expression level of each exon of a gene individually, which enables to identify alternative splicing events. In this array platform, all isoforms of a gene are combined to be called a ‘transcript cluster’, and each exon of the transcript cluster is defined as a ‘probe set’. In most cases, four probes are assigned to each probe set to measure the exon expression of a gene. By combining all probe sets, the expression of the transcript cluster, and therefore the gene, can be measured (15). In contrast to other microarray platforms, exon arrays contain many probes to expressed sequence tags (ESTs) and prediction-based transcripts (16). However, most exon array studies completely discard those additional probes and concentrate only on highly curated protein-coding genes (17–19); simply due to the fact that the analysis of such not-well-annotated probe sets is challenging both biologically and computationally.

Several studies show that microarrays can be re-annotated for lncRNAs. Michelhaugh *et al.* (20) found five lncRNAs to be up-regulated in heroin abusers using Affymetrix U133A and B microarrays. The same microarray platform was used to identify seven lncRNAs that are regulated in Huntington’s disease brains (21). Dinger *et al.* (22) used a large dataset of microarrays to build a database providing expression information for lncRNAs in human and mouse. GATExplorer (23) and ncFANs (24) re-annotated several microarrays from Affymetrix for protein coding and lncRNAs. Both tools provide files that can be used to process microarrays on a local computer for gene expressions of lncRNAs. In addition, ncFANs set up a web server for processing microarrays and creating a functional annotation of lncRNAs. While both tools support different types of Affymetrix microarrays, they report that exon arrays contain far more probes matching lncRNAs than any other microarray platforms.

In this report, we introduce an advanced annotation for exon arrays, which is the first one of its kind to measure both gene (that is, non-coding gene) and exon expressions of lncRNAs. To achieve this, we built the annotation in a similar way to the original Affymetrix annotation. Overlapping lncRNAs were combined to transcript cluster, and probe sets were defined to measure exon expressions. This enables a much more detailed inspection of lncRNAs than any other currently available annotations and software/databases that use such annotations. With our new annotation, it is possible to distinguish between overlapping transcripts and to identify alternative spliced lncRNAs.

In addition to the lncRNA annotation, we built an easy-to-use web interface called ‘noncoder’ (http://noncoder.mpi-bn.mpg.de). Noncoder includes all features of our previously introduced web interfaces ‘Exon Array Analyzer (EAA)’ (25) and ‘Gene Array Analyzer (GAA)’ (26). Furthermore, it can additionally pre-process raw exon array data with our custom annotation. A graphical presentation of gene and exon signals, an integrated genome browser and detailed information about lncRNAs allow for a practical and detailed examination of lncRNAs with ease.

## MATERIALS AND METHODS

### Building a custom lncRNA annotation for exon arrays

The 25-nt probe sequences of human and mouse exon arrays were downloaded from the manufacturer’s website (http://www.affymetrix.com). Genomic positions of each probe were identified by mapping them to the genome (hg19 and mm9) by bowtie (27). No mismatches were allowed and sequences mapping to none or multiple locations were discarded. Probes overlapping with protein-coding genes were excluded from further processing. For this purpose, we downloaded transcript annotations from ENSEMBL, UCSC (known gene track) and RefSeq (by UCSC’s table browser) with a protein identifier or defined coding sequence region. All probes that overlap on the same strand with exons from any of these annotations were excluded using BEDTools (28).

As a source of lncRNAs, we used the NONCODE3 database (29), which is a comprehensive collection of lncRNAs combining different sources of information about lncRNAs. We downloaded all sequences from human and mouse and discarded non-coding transcripts of 200 nt or shorter. Genomic positions of all sequences were obtained by a local BLAT server (30). Ambiguously mapped sequences were deleted, resulting in 33 909 and 37 266 lncRNAs for human and mouse, respectively. For each lncRNA, we used the Coding Potential Calculator (31) and PhyloCSF (32) to predict the non-coding character. For PhyloCSF, the conservation scores of 29 mammals for each transcript were downloaded using the ‘Stitch Gene blocks’ function on Galaxy (33). We used the Vienna RNA package (34) to predict the secondary structure of each lncRNA, which can be used for, e.g. designing effective siRNAs (35). Secondary structures and results from the prediction algorithms were imported into MySQL tables together with genomic positions and NONCODE3 annotations.

To generate an annotation that can measure gene and exon expressions separately, lncRNAs that overlap each other on the same strand were combined to ‘transcript cluster’. Then, ‘probe sets’ were defined as contiguous regions within the transcript clusters, meaning that a probe set is a region that is not interrupted by exon boundaries of any transcript in the transcript cluster. Exon array probes were assigned to the probe sets by their genomic positions. Probe sets without probes were deleted. All operations were conducted by BEDTools (28), Tabix (36) and custom Perl scripts.

For pre-processing of exon array CEL files, we use the Affymetrix Power Tools (APTs) (http://www.affymetrix.com). This tool requires various files containing information about the location of the probes on the chip, which probes should be used to estimate the background noise and which probes have to be clustered to probe sets and transcript cluster. We modified the original files from Affymetrix for our custom-defined probe sets and transcript clusters. All background probes were adopted. A schematic overview of the annotation process is shown in Supplementary Figure S1. The generated files were deposited on the noncoder web server for processing of user uploaded CEL files. For local processing, the annotation files are available for download as Supplementary Datasets S1 (human) and S2 (mouse) and on the noncoder website.

### Implementation of noncoder

The noncoder web interface is mainly written in PHP5. To provide an intuitive user experience, AJAX and jQuery, a client-side JavaScript library, have been used. The genome browser uses Perl libraries from the Bioperl toolkit (37). User data and annotations are stored in MySQL databases.

Pre-processing of microarrays is conducted by APT and further processing by Perl scripts and R for differential expression analysis by limma (38). For the exon-level analysis, noncoder uses the same approach as our previously introduced web server [see (25) and (26) for details]. Essentially, noncoder calculates the gene-level normalized intensities (GNIs) for each probe set. This value indicates the difference of the exon expression compared with the expression of the gene. The Splice Index (SI) is the logarithmic fold change of the GNIs between two groups and shows whether an exon is differentially expressed or not (39).

### GATExplorer and ncFANs

R packages with CDF files were downloaded from the websites of GATExplorer (23) and ncFANs (24). Using these annotations, exon arrays of murine heart and kidney were normalized with RMA (40) in R. Differentially expressed genes were identified by limma (38). For the direct comparison, the FANTOM3 accessions from the CDF file of ncFANs were converted to GenBank accessions by utilizing the following file: ftp://fantom.gsc.riken.jp/FANTOM3/DDBJ/DDBJ_fantom3_HTC_accession.txt.gz.

### RNA-Seq

Aligned RNA-Seq reads from mouse heart and kidney were downloaded from the ‘LICR RNA-Seq’ track of the UCSC genome browser. RNA was sequenced after poly-A+ purification in two replicates using a strand-specific protocol (41). Gene expression differences were calculated by cuffdiff (42). For protein-coding genes, the annotation from UCSC was used. For lncRNAs, we used our defined transcript clusters as annotation. Genes with <10 alignments in both tissues and genes with no alignment in either tissue were not used for the comparison.

### Hierarchical clustering

The normalized expression files were loaded into MeV (43) and clustered by Pearson’s correlation with average linkage clustering. For the gene-based cluster, MeV was used to filter for the 150 lncRNAs with highest variations. Clusters are generated by *k*-means with a cluster size of 5.

### Reverse transcriptase–polymerase chain reaction

RNA preparation, first-strand complementary DNA (cDNA) synthesis (using random primers) and reverse transcriptase–polymerase chain reaction (RT–PCR) experiments were performed as described previously (44).

## RESULTS

### LncRNA annotation

**Figure 1.**
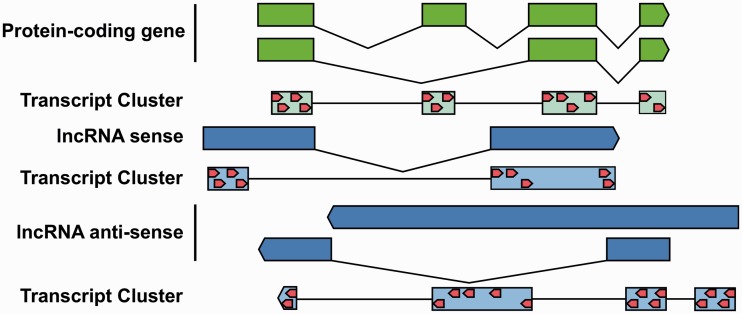
The Affymetrix annotation combines all isoforms of protein-coding genes (green) into a transcript cluster (light green). To measure the gene expression, all probes (red) of the transcript cluster are used. The exon expression can be measured by the probe set (light green boxes). We adopted this system for lncRNAs. Additional probes, which do not overlap with protein-coding genes, were used to define custom transcript cluster and probe sets. Overlapping lncRNAs on the same strand were combined to transcript cluster. Probe sets were defined to be contiguous regions not crossing exon boundaries. This allows for measuring gene and exon expressions, which are analogous to those of the Affymetrix annotation.

Exon arrays contain probes distributed across the entire length of a gene. To measure the gene and exon expressions, the Affymetrix annotation is organized into two different levels: the gene-level annotation to measure the gene expressions and the exon level to measure expression of exons. To achieve this, all isoforms of a gene are combined into transcript clusters. In this way, the gene expression is not measured by a single transcript but by the expressions of all transcripts in the cluster. At the exon level, each exon is covered by a probe set, resulting in expression signals for each exon of the transcript cluster (Figure 1).

We inspected all lncRNAs from the NONCODE3 database and found that the majority (87% in human and 67% in mouse) of the transcripts have more than one exon. The NONCODE3 database does not combine overlapping transcripts to genes, as it is common for protein-coding transcripts. Therefore, we clustered all lncRNAs to find out if the dataset contains overlapping and alternative spliced transcripts. For human, this resulted in 21 161 transcript clusters from originally 33 903 lncRNAs. For mouse, 37 266 lncRNAs were combined into 29 804 transcript clusters. This observation led us to the assumption that a lncRNA annotation can be built in a similar way to the original Affymetrix annotation.

**Figure 2.**
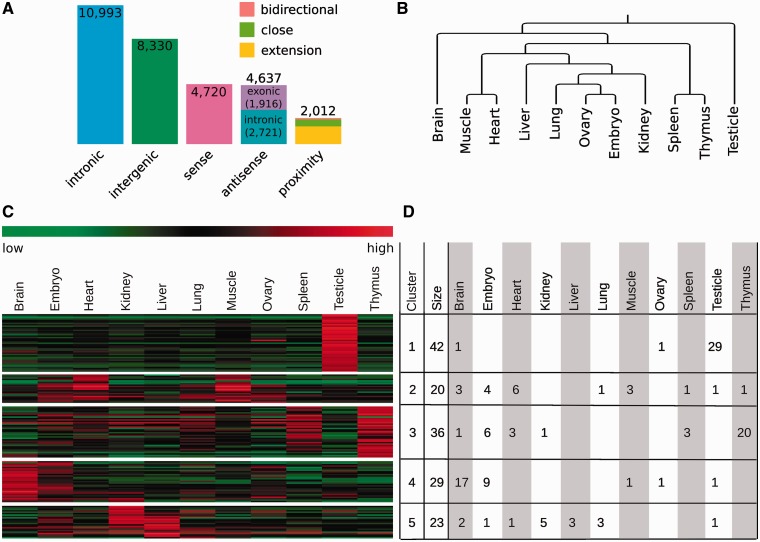
(**A**) Position of mouse lncRNAs in respect to protein-coding genes from ENSEMBL. Proximate was defined as closer than 1000 bp, but non-overlapping, to the next protein-coding gene. This category intersects into extension (encoded on the same strand, 542), close (opposite strand, 1404) and bidirectional (opposite strand in head-to-head orientation, 66). (**B**) Dendrogram generated from lncRNA gene expressions of 11 murine tissues. (**C**) Heat map of the 150 most differentially expressed lncRNAs across 11 different murine tissues. For *k*-means clustering, we used *K* = 5. (**D**) For each lncRNA with GenBank accession number, we looked up the tissue where the cDNA has been originally found. All embryonic libraries have been classified as ‘embryo’, and ‘tongue’ as ‘muscle’ and ‘aorta and vein’ as ‘heart’.

To ensure that the expression measurement of lncRNAs is not biased by probes mapping to protein-coding genes, we removed all probes for protein-coding genes completely. This also removes lncRNAs that entirely overlap with protein-coding genes and lncRNAs to which no unique probes are on the exon array. Based on the remaining lncRNA transcript clusters, we defined our own probe sets (Table 1). These are regions that are not intersected by exon boundaries from any transcripts in the lncRNA cluster and are covered by exon array probes (Figure 1). The coverage by probes of the total length of lncRNAs is lower than that of protein-coding genes (8.6% compared with 29%; Supplementary Figure S2). For each of these lncRNAs, we calculated the distances to neighbouring protein-coding genes and classified them into seven categories (Figure 2A).

**Table 1.** Overview of the lncRNA annotation

	Human	Mouse
Total probes	4 996 300	4 406 421
Protein-coding probes	1 328 113	1 168 746
Probes matching to lncRNAs (not protein-coding genes)	155 485	264 382
Probe sets	34 315	49 216
Transcript clusters	12 007	24 607
lncRNA transcripts	21 681	30 692

All probes of the human and mouse exon arrays were mapped to the genome. Probes mapping to protein-coding genes were discarded for further analysis. Only the remaining probes were mapped to lncRNAs. Based on these filtering steps, we defined probe sets and transcript clusters.

For an initial test of our custom lncRNA annotation at the gene level, we applied it to a publicly available exon array dataset (http://www.affymetrix.com) of 11 different mouse tissues. The results of hierarchical clustering shows that related tissues (e.g. heart and muscle) share similar expression patterns of lncRNAs, whereas brain and testicle express a distinct set of lncRNAs (Figure 2B). This indicates that like protein-coding genes, lncRNAs show tissue-restricted expression patterns. To understand the clustered results better, we filtered out 150 lncRNAs with the most variation across all samples and clustered by their gene expressions (Figure 2C). For each of these lncRNAs, we looked up the GenBank description to find out in which tissue the cDNA has been identified originally (Figure 2D). This information allows us to check if the expression pattern of the clusters is reflected by the tissues the lncRNAs were found in. For the clusters with high expression in testicle, brain and thymus, we found the occurrences of 93.5, 58.8 and 58.6%, respectively.

### Noncoder

The noncoder is an advancement of our previously introduced web interfaces EAA (25) and GAA (26). It combines the analysis of exon arrays and Affymetrix GeneChip Gene 1.0 ST arrays (gene arrays) for protein-coding genes and implements the same features as the original web interfaces. Additionally, the user interface has been completely re-designed for a better user experience. One main feature is the possibility to create an account, where microarrays can be deposited. This allows analysing the microarrays using different parameters without uploading the files again. Further, we implemented an option to import microarrys directly from ArrayExpress. This can be of use to re-analyse published studies for lncRNAs.

**Figure 3.**
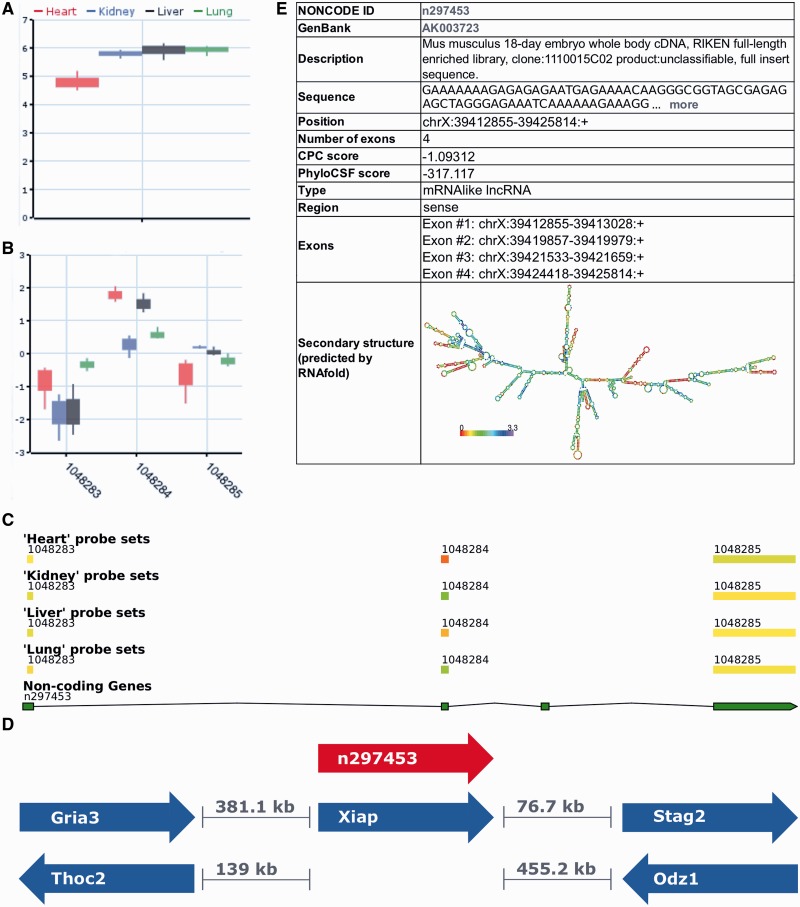
Features of noncoder. Noncoder provides various features: (**A**) Box-and-Whisker plot of transcript cluster and (**B**) GNIs. This lncRNA is not differentially expressed between the kidney and liver, but a probe set has a high GNI difference. (**C**) The graphical representation of the same lncRNA as above in our built-in genome browser. A probe set with the high difference in GNI is coloured to show lower expression in the kidney (green) than liver (orange). (**D**) Noncoder provides a schematic overview of the location of the lncRNA to other genes. (**E**) A table with the detailed annotation information about each lncRNA of a cluster. Besides the genomic location and GenBank description, it shows the non-coding prediction score (from CPC and PhyloCSF), the location to protein-coding genes (‘Region’) and predicted secondary structure.

For the analysis of exon arrays for lncRNAs, we implemented several additional features. First, our custom annotation files are used to pre-process exon arrays. This is done by default for all exon array analyses; thus, the user can obtain results for both protein-coding genes and lncRNAs. When the analysis has been completed, the user can access the result and filter for differentially expressed genes and exons or filter by NONCODE3, GenBank ID or position to protein-coding genes. Each transcript cluster is linked to a web page with the detailed information about the expression and annotation of lncRNAs in the cluster. This page is divided into the following four tabs: The ‘Expression’ tab shows the expression differences of the analysed groups at the gene (Figure 3A) and exon levels (Figure 3B). On the same page, the integrated genome browser of noncoder shows the genomic position of the lncRNAs together with probe sets (Figure 3C). This allows to inspect alternative splice events and to distinguish between expressed and non-expressed isoforms of the cluster. Additionally, the genome browser includes additional gene annotations from UCSC, ENSEMBL, AceView and EST based transcripts. For a more detailed view, we provide a link to the UCSC genome browser. We generated custom tracks that show the NONCODE3 and our custom exon array annotation. The ‘Transcripts’ tab shows information about all lncRNA members of the current cluster (Figure 3E), including the genomic position, sequence and score from the non-coding prediction results. Under the ‘Array mapping’ tab, all probe sets are listed together with their genomic position and sequence. ‘Nearby genes’ tab shows the relative position of the lncRNAs to nearby protein-coding genes (Figure 3D). Genes are linked to the result page of the analysis for protein-coding genes for more detailed inspection.

A full manual of noncoder with additional information can be found on its website. The noncoder is available at http://noncoder.mpi-bn.mpg.de and can be used with or without registration and is free of charge.

### Performance of noncoder compared with other annotations

**Figure 4.**
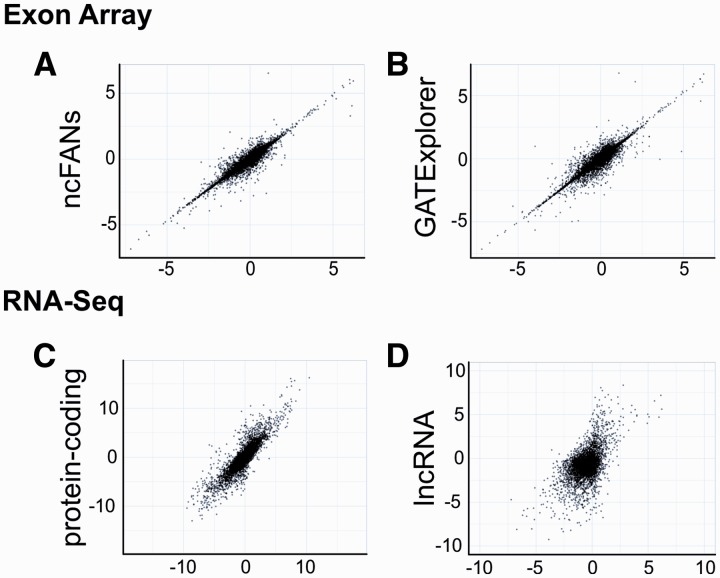
Correlation of the log_2_ fold changes between the heart and the kidney of the (**A**) ncFNAs and (**B**) GATExplorer annotations compared with our lncRNA annotation (*x*-axis). The Pearson’s correlations are 0.95 and 0.96, respectively. (**C**) Correlation of the fold changes of protein-coding genes between the heart and kidney measured by RNA-Seq and noncoder (*x*-axis). The Pearson correlation is 0.87. (**D**) Correlation of lncRNAs. The Pearson correlation is 0.55.

**Figure 5.**
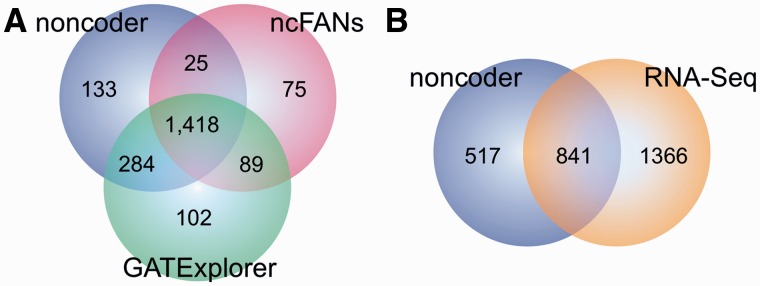
(**A**) Differentially expressed lncRNAs between the heart and the kidney (log_2_ fold change <−1 or >1) detected by noncoder, GATExplorer and ncFANs. (**B**) Differentially expressed lncRNAs detected by noncoder and RNA-Seq. For RNA-Seq, we used our lncRNA annotation as reference. Only lncRNAs that have >10 reads in the heart or kidney and >0 reads in either tissue were used.

GATExplorer and ncFANs provide lncRNA annotations for exon arrays. ncFANs has been written by the same authors who built the NONCODE3 database; however, only lncRNAs from the FANTOM3 project were included. Both annotations were built to measure expression levels of transcripts. Compared with the above two tools, our annotation is based on a different approach as it measures transcript cluster and probe sets. For a comparison, we pre-processed exon arrays from murine heart and kidney with noncoder and by using annotation files provided by GATExplorer and ncFANs. A side-by-side table can be found in Supplementary Table S1. Fold changes between the heart and kidney show high correlations between the annotations (Figure 4A and B). The overlap of differentially expressed lncRNAs is shown in Figure 5A. The lncRNA with the largest fold change difference is AK085889. GATExplorer and ncFANs show an up-regulation of over 6-fold in the heart, while noncoder shows a significant (*P*-value 0.0036) but lower fold change of 1.1 (fold changes in base 2 logarithm scale). The transcript cluster, which contains AK085889, combines 21 overlapping lncRNAs. Only a part of the transcript cluster probes show differential expressions, which explains the lower fold change computed by noncoder. Furthermore, the probe set expressions reveal that a larger region than the one covered by AK085889 is up-regulated in the heart (Supplementary Figure S3). This indicates that the overlapping transcript AK085765 is also expressed higher in the heart. RT–PCR validation confirms this observation (see below). GATExplorer and ncFANs do not contain AK085765 in their annotations, which confirms the novelty of our annotation and the applicability of noncoder.

### Comparison to RNA-Seq

Since no *a priori* knowledge about the transcriptome is required, RNA-Seq has the potential to detect all expressed transcripts. To benchmark our lncRNA annotation, we compared it with the results of RNA-Seq data, which are publicly available, for the heart and kidney. We compared the fold changes between the heart and kidney for protein-coding genes and lncRNAs. For a direct comparison, we used only the protein-coding genes that are present in the UCSC annotation and the original Affymetrix annotation (Figure 4C). For lncRNAs, we used our defined transcript cluster and considered only those reads that map within these regions (Figure 4C; for a side-by-side table, see Supplementary Table S2). A comparison of differentially expressed lncRNAs detected by both techniques is shown in Figure 5B. In this comparison, the sources of biological tissues differ significantly as well as other biological and technical biases common to both techniques. Nevertheless, an overlap of 38.10% (841/2207 lncRNAs identified by RNA-Seq) between our noncoder to the results of RNA-Seq was observed. This indicates that our annotation can be used to record the expression changes of lncRNAs. It should be added to note that no biological (e.g. RT–PCR, *in situ* hybridization) large-scale validations of expression changes of tissue-specific lncRNAs have been done in the past; thus, 517 lncRNAs identified only by our noncoder could also count for novel tissue-specific lncRNAs that could not be detected to be statistically significant by RNA-Seq.

### Validation by RT–PCR

To validate the potential of noncoder to detect differentially expressed lncRNAs, we performed RT–PCR of example candidates. For this purpose, we analysed exon arrays of heart, kidney, liver and lung from the tissue dataset with noncoder using default parameters. A list of differentially expressed lncRNAs can be found in Supplementary Table S3 and on the noncoder website. Since alternative splicing events can be sex-specific (45), we prepared cDNA from male and female mice separately to address this point as well for lncRNAs.

**Figure 6.**
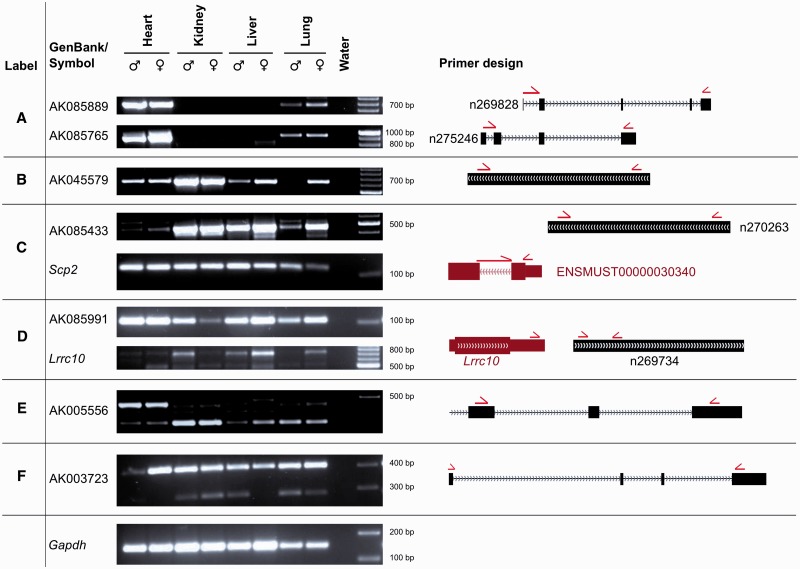
To validate lncRNA expression changes, we used cDNA of the heart, kidney, liver and lung from male and female mice. The location of the primers in respect to lncRNAs (black) and protein-coding genes (red) is shown on the right column. Primers spanning exon–exon junctions are displayed, which are stretched across both exons. The primer sequences can be found in Supplementary Table S4. (**A**) AK085889 has the highest fold-change between the heart and kidney according to ncFANs and GATExplorer annotations. Due to our annotation, which is designed to measure exons individually, we could find out that the overlapping lncRNA AK085765 is also up-regulated in the heart. (**B**) The expression of AK045579 is up-regulated in the kidney according to noncoder annotation, while GATExplorer shows lower expression in the kidney compared with the other three tissues. Of note, sex difference in the expression of this lncRNA can be recorded in the lung. (**C**) AK085433 overlaps with the protein-coding gene *Scp2* and is encoded on the same strand. Noncoder measured the expression of the lncRNA to be high in the kidney compared with the other tissues. RT–PCR result shows strong bands in the kidney but also in the liver (∼454 bp). To ensure that the lncRNA is not part of the protein-coding gene *Scp2*, another primer pair was designed (to the isoform ENSMUST00000030340 of *Scp2*). In contrast to the lncRNA, the expression level of *Scp2* is similar in the heart, kidney, liver and slightly lower in the lung. (**D**) AK085991 shows the highest expression in the heart compared with the kidney according to noncoder. This transcript is in close distance to *Lrrc10* and encoded on the same strand. Another forward primer was used to detect if the lncRNA is connected to *Lrrc10*, but only weak bands are visible (∼653 bp). (**E**) Noncoder shows an up-regulation of the sixth exon of AK005556 in the heart, which is in accordance to the RT–PCR result. (**F**) The second exon of AK003723 is lowly expressed in the kidney and lung compared with the heart and liver. RT–PCR shows the longer isoform expressed in all tissues (∼406 bp) and the shorter one (∼283 bp) in the kidney and lung as computed by noncoder but also in male liver.

First, we tested the expression of lncRNAs that show different fold changes in comparison to GATExplorer and ncFANs. As described above, the transcript AK085889 shows a lower fold change using noncoder when comparing the heart with the kidney. The result of the RT–PCR experiment shows that this transcript is indeed highly expressed in the heart. However, RT–PCR shows that the overlapping transcript AK085765 is also highly expressed in the heart (Figure 6A), which is not part of the GATExplorer or ncFANs annotations. Furthermore, we tested the lncRNA AK045579, which is the second highest difference in its expression between GATExplorer and noncoder. While GATExplorer predicts an up-regulation in the heart compared with the other three tissues, noncoder shows up-regulation in the kidney, which we confirmed by RT–PCR (Figure 6B).

The lncRNAs AK085433 and AK085991 are regulated as a result of noncoder. AK085991 shows the highest fold change in the heart compared with the kidney. Both are in close distance to protein-coding genes, which are encoded on the same strand. With additional primer pairs, we show that the lncRNA expression alters as shown by the noncoder but is independent of the proximate protein-coding gene (Figure 6C and D).

In addition, two alternative spliced lncRNAs were validated. AK005556 and AK003723 show high SIs for some probe sets, indicating exon skipping events. We designed primers on the flanking exons and observed the expected band sizes (Figure 6E and F). Both splice events are not part of the NONCODE3 annotation.

The primer sequences used for the RT–PCRs can be found in Supplementary Table S4.

## DISCUSSION

Exon arrays are widely used to profile gene expressions and alternative splicing events. Here, we present a new application for exon arrays by building a custom annotation to measure lncRNAs. In contrast to other custom annotations, we built it in a similar way to the original Affymetrix annotation to measure the expression levels of lncRNAs and their individual exons. This scheme enables a much more detailed inspection of their expressions. Our easy-to-use web server called ‘noncoder’ has been built to support the user with such analysis. It can process exon arrays for protein-coding genes and lncRNAs. Various features, such as visualization of the probe set expressions and an integrated genome browser, make the noncoder a valuable tool to analyse exon arrays for lncRNAs.

Initially, we tested our lncRNA annotation by analysing a dataset of 11 murine tissues. By clustering the expression values, we showed that lncRNAs are expressed in a tissue-specific manner. This was validated by the GenBank annotations of the 150 most differentially expressed lncRNAs across these tissues and shows that exon arrays have the potential to measure lncRNAs.

For gene-level expressions of lncRNAs, other custom annotations have been built for various microarrays, including exon arrays from Affymetrix (20–24). In this study, we concentrated on exon arrays because this array platform greatly exceeds other microarrays for the coverage of lncRNAs (23). To allow for an efficient annotation and further analysis, we built a lncRNA annotation in a fashion similar to the Affymetrix annotation for protein-coding genes. Overlapping lncRNAs were clustered together, and each exon of such a transcript cluster has been defined as a probe set to measure exon expressions. Although 155 485 and 264 382 lncRNAs are represented in human and mouse exon arrays, respectively, a majority of probes is not mapping to lncRNAs (Table 1). A part of reasons can be that the current annotation of lncRNAs is incomplete, which is evident from the fact that such annotation of lncRNAs is permanently increasing as more and more biological data (e.g. RNA-Seq) are generated. To cope with this increasing number of new lncRNAs, noncoder will be updated regularly with the release of new annotation files in NONCODE database.

We analysed and compared the same exon array datasets with lncRNA annotations from GATExplorer and ncFANs to compare the results with each other. We found that the correlation at the gene level is very high between the different annotations. However, as in the case of AK085889, the fold change of a transcript cluster can be less severe due to the combination of all overlapping transcripts. Using the exon-level annotation of this transcript cluster revealed that another overlapping transcript is also regulated. This finding was not identified by the other annotations and demonstrates the advantage of our lncRNA annotation. To add simply, we biologically validated such observations by performing RT–PCR experiments, while others did not do so.

We also compared the exon array results with RNA-Seq data to see whether the measurements of lncRNAs are comparable. Sequencing has been conducted using a strand-specific protocol, which is not default for Illumina sequencing. Without this protocol, it would not have been possible to distinguish between protein-coding genes and their anti-sense lncRNAs. We note that exon array and RNA-Seq data have been obtained from different public sources, which might reflect in different expression profiles. Further, the protocols, which have been used for sample preparation, differ: RNA-Seq used poly-A selection, while exon array samples are prepared by random hexamer primers. Since it has been reported that a high number of lncRNAs do not undergo polyadenylation (6), exon arrays might also be a valuable tool to study these transcripts. However, this has not been further evaluated here. Despite the differences of the techniques and samples, we found a high correlation between RNA-Seq and exon arrays for protein-coding genes, which is comparable to the findings of others (46,47). The correlation for our lncRNA annotation was lower (*r* = 0.55) but still significant (*P*-value < 2.2e−16). Reasons for the lower correlation might be the less and not optimal coverage of probes on the exon arrays for lncRNAs. Also, microarrays do not perform well for lowly expressed genes when compared with those with higher expressions (46,48). The general low expression of lncRNAs might also be of an issue for RNA-Seq. Less abundant transcripts might not be covered by enough reads for a reliable detection (Supplementary Figure S4) and high sequencing depth can still be expensive (49). Overall, these observations lead to the conclusion that exon arrays are applicable for measuring lncRNAs in a way comparable to RNA-Seq.

Exon arrays are almost exclusively used to analyse gene and exon expression changes of protein-coding genes. In this study, we show that probes on exon arrays can be used to measure expression changes of lncRNAs. We set up a custom annotation that is specifically designed for this purpose. Using this custom annotation, we identified and validated expression changes and alternative splicing events of lncRNAs. To make such an analysis as easy as possible, we provide a web interface that analyses exon arrays with our custom annotation. It is designed to require no knowledge on programming. The features (e.g. information about lncRNAs, visualization of the gene and exon expressions and an integrated genome browser) support the users to identify differentially expressed lncRNA. This enables a broad range of researchers to analyse their already existing and prospective exon array experiments from the perspective of lncRNAs.

## AUTHORS CONTRIBUTIONS

S.U. and P.G. conceptualized the study. P.G. developed noncoder, the lncRNA annotation and performed computational analysis. Y.P. performed biological experiments. T.B. provided general guidance. S.U. supervised the project. The manuscript was prepared by P.G. and S.U. with inputs from other co-authors. All authors read and approved the final manuscript.

## SUPPLEMENTARY DATA

Supplementary Data are available at NAR Online: Supplementary Tables 1–4, Supplementary Figure 1–4 and Supplementary Datasets 1 and 2.

## FUNDING

LOEWE Center for Cell and Gene Therapy Frankfurt (CGT) [‘Hessian Ministry of Higher Education, Research and the Arts’ funding reference number: III L 4-518/17.004 (2010) to S.U.]; Excellent Cluster Cardio-Pulmonary System (ECCPS) (to S.U. and T.B.); LOEWE Center for Cell and Gene Therapy (UGMLC) (to S.U. and T.B.); Max-Planck-Society and DFG (Br1416) (to T.B.). Funding for open access charge: Max-Planck-Society.

*Conflict of interest statement*. None declared.

## Supplementary Material

Supplementary Data
