# Supplementary material for: Noncoder: a web interface for exon array-based detection of long non-coding RNAs
Source: Nucleic Acids Res. 2012 Sep 24;41(1):e20. doi: 10.1093/nar/gks877 (PMC3592461; doi:10.1093/nar/gks877)
Supplement: Supplementary Data [file supp_41_1_e20__index.html]

Noncoder: a web interface for exon array-based detection of long non-coding RNAs — Supplementary Data 

# Noncoder: a web interface for exon array-based detection of long non-coding RNAs

## Supplementary Data

files

**Files in this Data Supplement:**

- Supplementary Data - pdf file
- Supplementary Data - zip file
- Supplementary Data - zip file
- Supplementary Data - xls file
- Supplementary Data - xls file
- Supplementary Data - xls file
- Supplementary Data - xls file
