# Supplementary material for: Noncoder: a web interface for exon array-based detection of long non-coding RNAs
Source: Nucleic Acids Res. 2012 Sep 24;41(1):e20. doi: 10.1093/nar/gks877 (PMC3592461; doi:10.1093/nar/gks877)
Supplement: Supplementary Data [file supp_gks877_Gellert_Supplementary_Figures.pdf]

Supplementary Table S1:
Direct comparison of lncRNA expressions from noncoder, GATExplorer (21) and ncFANs (22). Only those lncRNAs that are present in noncoder and the compared annotation are shown. Exon arrays of murine heart and kidney were normalized with RMA using noncoder and the CDF files from the websites of GATExplorer and ncFANs.

Supplementary Table S2:
Direct comparison results from noncoder to those of RNA-Seq data. The RNA-Seq data of murine heart and kidney were downloaded from the UCSC genome browser (LICR RNA-seq track). Fold changes and p-values were calculated by cuffdiff (40). As a reference annotation for cuffdiff, we used UCSC's known genes track for protein-coding genes and our custom annotation for lncRNAs.

Supplementary Table S3:
Exon arrays of murine heart, kidney, liver and lung were analysed by noncoder using default parameters. All four tissues were compared with each other. The table shows fold changes and p-values at the levels of genes and exons as generated by noncoder.

Supplementary Table S4:
Primer sequences used for RT-PCR validation in Figure 6.

Supplementary Dataset S1 & S2:
Archives containing files for human (S1) and mouse (S2) to pre-process exon arrays at the level of lncRNAs. The following command is an example using the Affymetrix Power Tools (www.affymetrix.com)

```
apt-probeset-summarize -a rma-bg,quant-norm.sketch=-
1.bioc=true.lowprecision=false.usepm=true,pm-only,med-polish -a
pm-only,dabg.chisq=true \
-p MoEx-ncRNA.pgf -c MoEx-1_0-st-v1.r2.clf -b MoEx-
ncRNA.antigenomic.bgp \
--qc-probesets MoEx-ncRNA.qcc -m MoEx-ncRNA.mps \
exonarray1.CEL exonarray2.CEL exonarrayN.CEL -o apt-result_gene
```

On exon level

```
apt-probeset-summarize -a rma-bg,quant-norm.sketch=-
1.bioc=true.lowprecision=false.usepm=true,pm-only,med-polish -a
pm-only,dabg.chisq=true \
-p MoEx-ncRNA.pgf -c MoEx-1_0-st-v1.r2.clf -b MoEx-
ncRNA.antigenomic.bgp \
--qc-probesets MoEx-ncRNA.qcc -s MoEx-ncRNA.ps \
exonarray1.CEL exonarray2.CEL exonarrayN.CEL -o apt-result_exon
```

The archives also contain annotation files at the levels of exons and genes, including genomic coordinates, NONOCODE3 ID, GenBank ID, GenBank description and location to protein-coding genes.

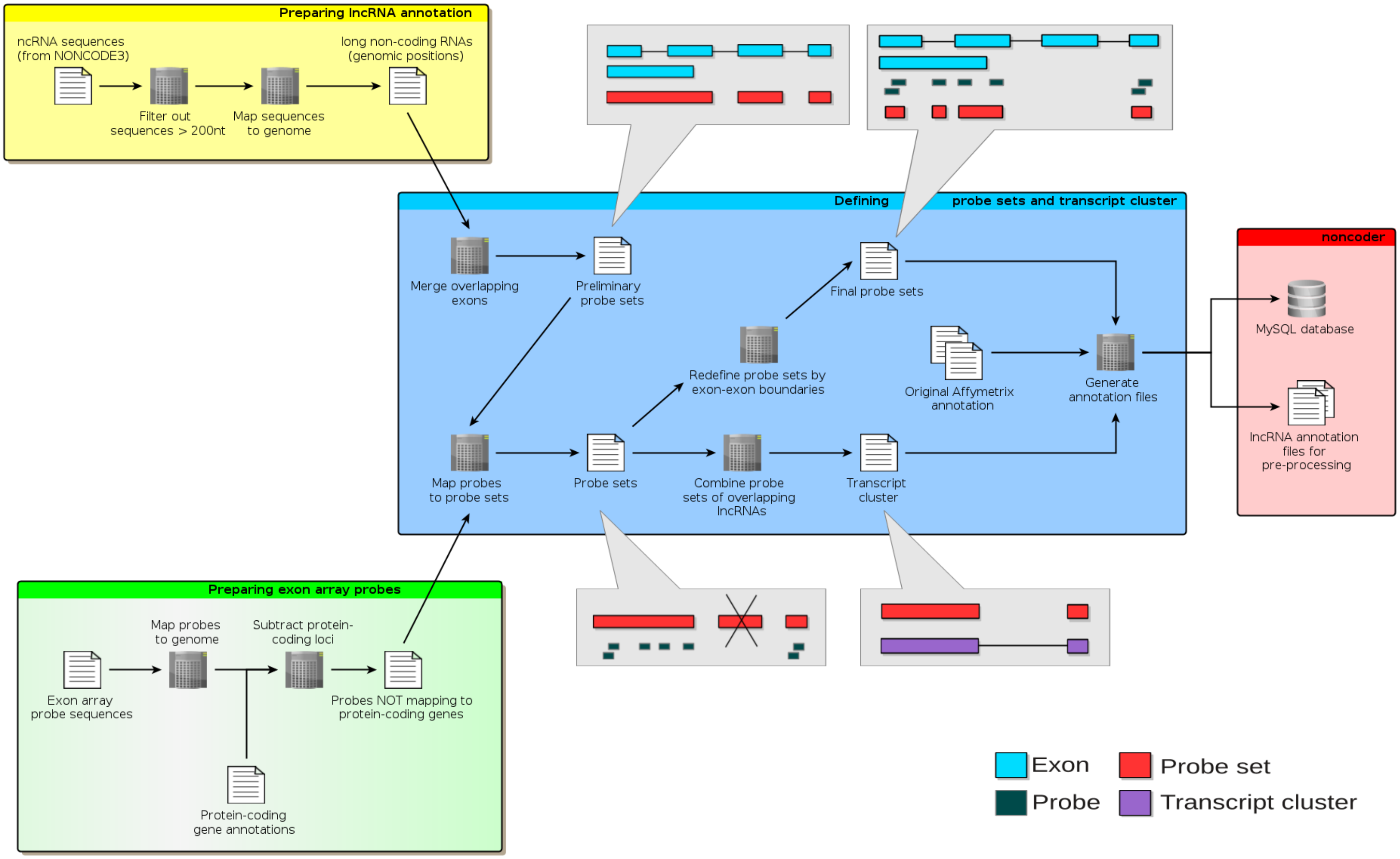

Supplementary Figure S1:
Simplified workflow of the lncRNA annotation process.

- Step 1 (green area):
  Exon array probes were prepared by aligning the 25nt sequences to the genome. Genomic positions were used to discard probes that map to protein-coding genes.
- Step2 (yellow area):
  Non-coding RNA sequences were downloaded from NONCODE3 (http://www.noncode.org/NONCODERv3/). This dataset contains short and long non-coding RNAs. Long non-coding RNAs were obtained by filtering for sequences larger than 200nt. Genomic positions of all lncRNAs were obtained by mapping each sequence to the genome using BLAT.
- Step 3 (blue area):
  Overlapping exons from Step 1 were combined to preliminary probe sets. Probes obtained from Step 2 were mapped to the preliminary probe sets. Probe sets to which no probes could be mapped were discarded. Based on this probe sets, transcript cluster can be defined by combining all probe sets of overlapping transcripts. Probe sets that are intersected by exon boundaries were separated. This results in final probe sets, which allow to measure each exon of a transcript cluster individually.
  The original Affymetrix files were used to generate custom annotation files for lncRNAs.
- Step 4 (red area):

The generated files were uploaded to the noncoder server for the pre-processing of exon arrays. Probe set and transcript cluster information were imported into MySQL tables for the noncoder web interface.

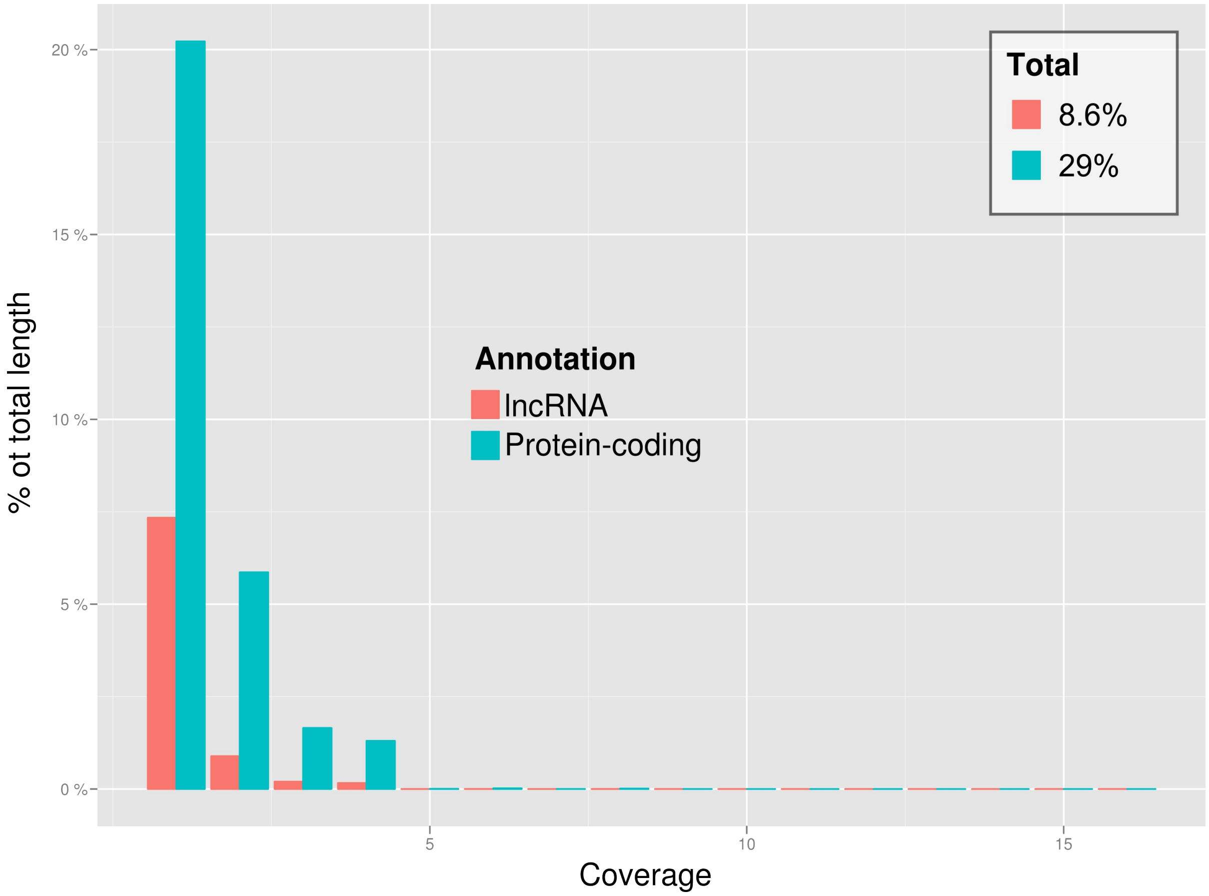

Supplementary Figure S2:
Comparison of the probe coverage for lncRNAs and protein-coding genes. This figure shows the percentage of the protein-coding genes and lncRNAs regions that are covered by 1 to 16 probes. In total, 29% of the regions spanned by protein-coding genes are covered by at least one probe. For lncRNA 8,6% are covered. 1.3% of the protein-coding genes and 0.17% of lncRNAs are covered by 3 or more probes.

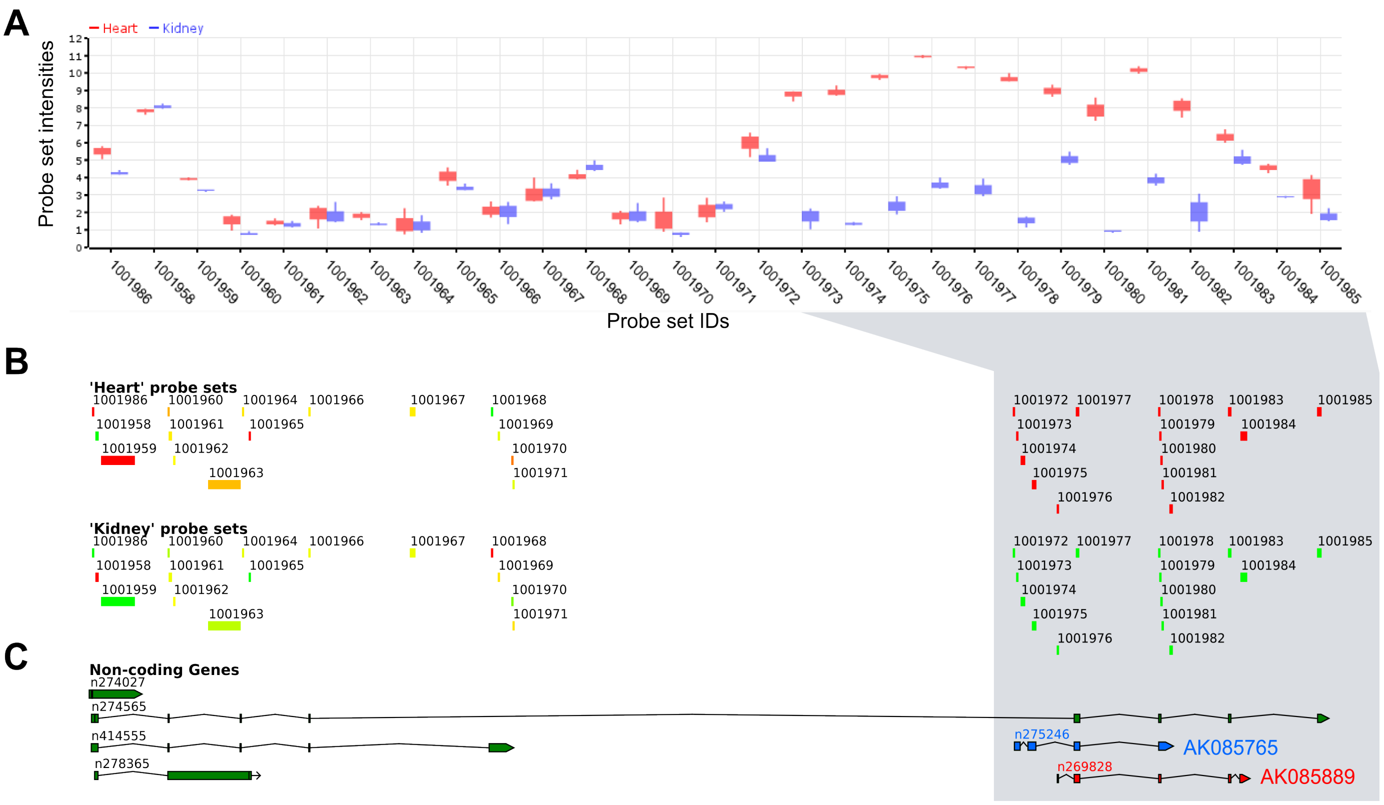

Supplementary Figure S3:
The lncRNA annotation from GATExplorer and ncFANs show AK085889 to be up-regulated over six-fold in the heart compared to kidney. Our own annotation combines this transcript together with other overlapping lncRNAs to a transcript cluster (C). The fold change of heart compared to kidney of the transcript cluster shows an up-regulation of 1.1. A closer look at the individual probe set expressions of the transcript cluster shows, that a larger region is up-regulated (A and B grey area). This indicates a higher transcription rate of the overlapping transcript AK085765 in the heart. This lncRNA is not contained in GATExplorer and ncFANs.

All graphics are modified from the output of noncoder. Additional lncRNAs have been deleted from (C) for a better overview.

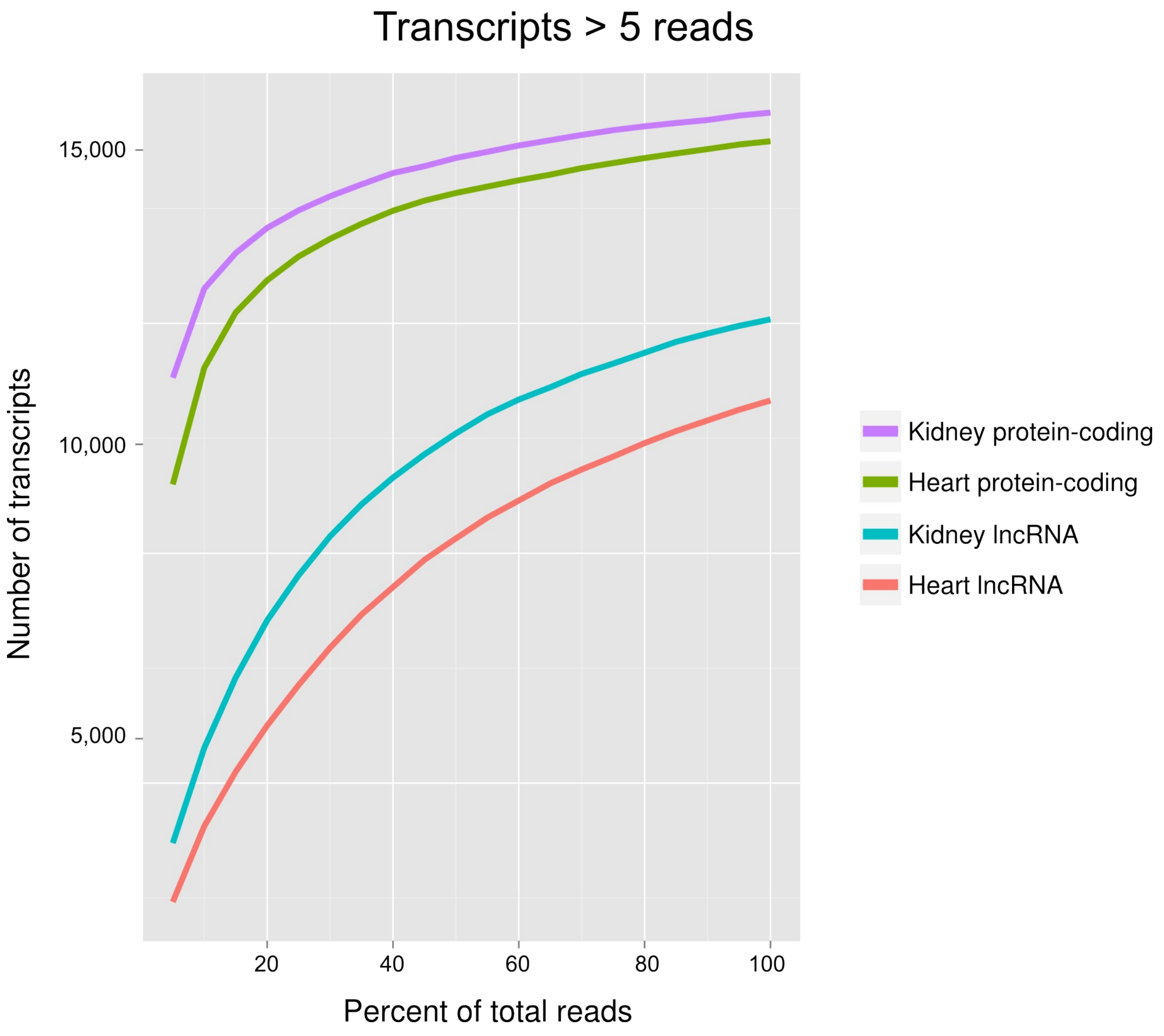

Supplementary Figure S4:
Total RNA reads from the heart and kidney RNA-Seq data were re-sampled using our lncRNA and UCSC's protein-coding annotation. The Figure shows the number of transcripts with more than 5 reads by using 5 to 100% of all reads. A saturated sequencing depth is reached, when the curves reach a plateau. The curves of lncRNAs in the heart and kidney (red and blue) do not reach a plateau as the curves for protein-coding genes, indicating that a higher sequencing depth is needed.
Re-sampling has been conducted by using RseQC (http://code.google.com/p/rseqc/).
